# Gallstone ileus with sigmoid impaction through a cholecystocolic fistula: a case report

**DOI:** 10.1093/jscr/rjaf1031

**Published:** 2026-04-25

**Authors:** Yves Lévesque, Corinne Brideau, Jessie Drouin

**Affiliations:** Department of General Surgery, Hôpital Hôtel-Dieu d’Arthabaska, Victoriaville, Université de Sherbrooke, QC G6P 6N2, Canada; Centre de formation médicale du Nouveau-Brunswick, 50 rue de la Francophonie, Moncton, NB E1A 7R1, Canada, Université de Sherbrooke, Moncton, NB, Canada; Centre de formation médicale du Nouveau-Brunswick, 50 rue de la Francophonie, Moncton, NB E1A 7R1, Canada, Université de Sherbrooke, Moncton, NB, Canada

**Keywords:** gallstone ileus, cholecystocolic fistula, biliary ileus, sigmoid impaction

## Abstract

Gallstone ileus is a rare cause of bowel obstruction. Colonic impaction, especially in the sigmoid colon, is extremely uncommon and often associated with diverticulosis or structural abnormalities. An 81-year-old woman presented with 2 weeks of abdominal pain, bloating, nausea, and vomiting. A computed tomography (CT) scan revealed a 6.2 cm gallstone in the sigmoid colon with pneumobilia and a cholecystocolonic fistula. After unsuccessful colonoscopic extraction, she underwent laparoscopic enterolithotomy with segmental sigmoid resection. She recovered without complications and remained symptom-free at her 6-week follow-up. Colonic gallstone ileus is rare and should be suspected in elderly women with obstruction and pneumobilia. A CT scan is the diagnostic gold standard. While endoscopy may work for small stones, surgery is usually required. Enterolithotomy with or without resection remains the safest approach in frail patients.

## Introduction

Gallstone ileus is an uncommon but clinically significant cause of mechanical bowel obstruction, accounting for <0.1% of all cases [1, 2]. It is the result of a gallstone entering the gastrointestinal tract through a cholecystoenteric fistula, most often into the duodenum or stomach, and less commonly into the colon [2]. Once in the intestinal lumen, the stone can migrate distally until it becomes lodged and causes obstruction. Gallstone ileus is more prevalent in elderly females, partly due to higher gallstone prevalence and age-related gastrointestinal and biliary changes [3]. Repeated cholecystitis can lead to cholecystocolonic fistula, most often at the hepatic flexure. Obstruction of the large bowel due to gallstone impaction in the sigmoid colon is rare and usually occurs at sites of pre-existing inflammation, diverticular disease, or other structural abnormalities. This condition often presents diagnostic and treatment challenges due to its gradual onset and nonspecific symptoms, leading to delayed diagnosis and increased morbidity and mortality [3]. Colonic gallstone ileus should be suspected in elderly patients presenting with symptoms of bowel obstruction, pneumobilia on imaging, and evidence of an ectopic gallstone [2]. In this article, we describe a case of gallstone ileus with sigmoid colon impaction through a cholecystocolic fistula, along with its diagnostic challenges and management strategies for this rare condition.

## Case report

An 81-year-old Caucasian female patient was admitted to the hospital with a 2-week history of abdominal pain, bloating, nausea, and vomiting. She also reported diarrhea the day before admission. Her medical history included hypertension, dyslipidemia, and atrial fibrillation with a CHA₂DS₂ score of 2, without anticoagulation. Surgical history was notable for hysterectomy, bilateral oophorectomy, salpingectomy, and an endoscopic retrograde cholangiopancreatography (ERCP) for choledocholithiasis in 2023.

On admission, her vital signs were as follows: blood pressure 144/80 mmHg, heart rate 91 beats per minute, respiratory rate 16 breaths per minute, and body temperature 37.1°C. On physical examination, the patient exhibited tenderness to palpation in the left lower quadrant, with decreased bowel sounds on auscultation. Murphy’s sign was negative, and there were no signs of peritonism. Laboratory studies revealed an elevated white blood cell count of 13 × 10^9^/l with neutrophil predominance, and an elevated C-reactive protein level of 44.79 mg/l. A computed tomography (CT) scan revealed a large gallstone (cholelithiasis) within the lumen of the proximal sigmoid colon, along with pneumobilia. A fistula was identified between the gallbladder and the hepatic flexure of the colon. The gallbladder wall appeared markedly thickened and irregular.

**Figure 1:**
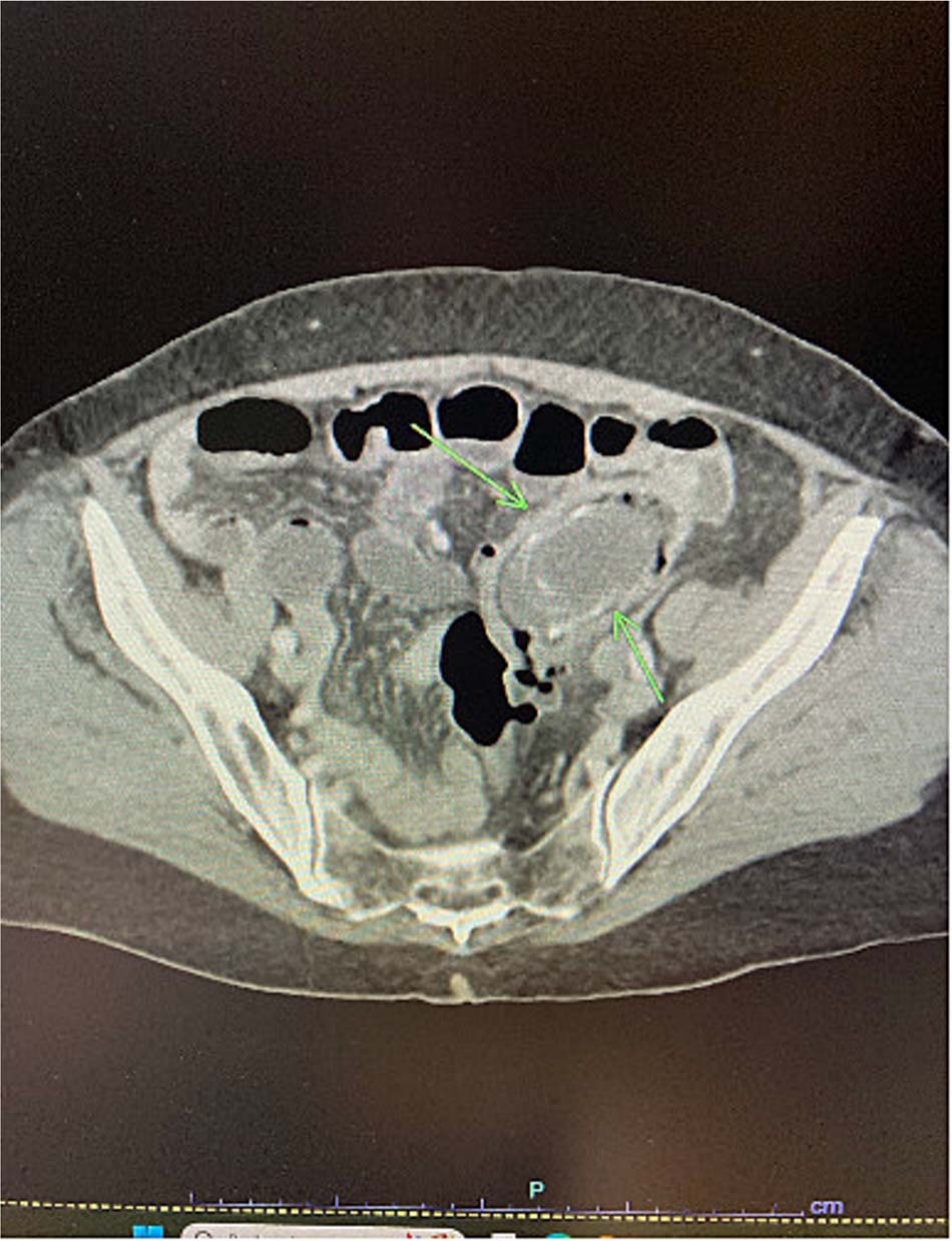
Arrows showing gallstone impacted in the proximal sigmoid colon on CT scan.

**Figure 2:**
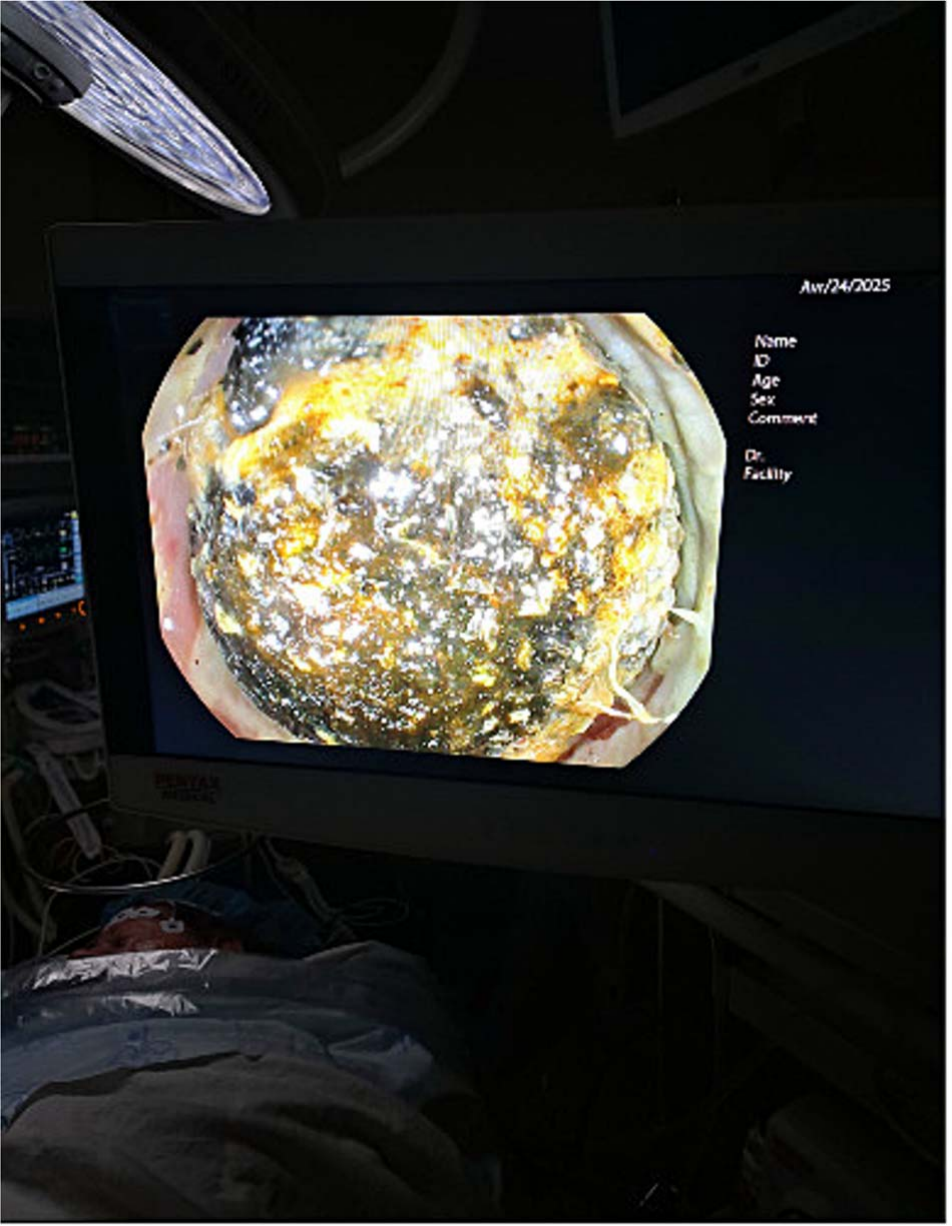
Gallstone located in the proximal impacted in the sigmoid colon identified on colonoscopy.

**Figure 3:**
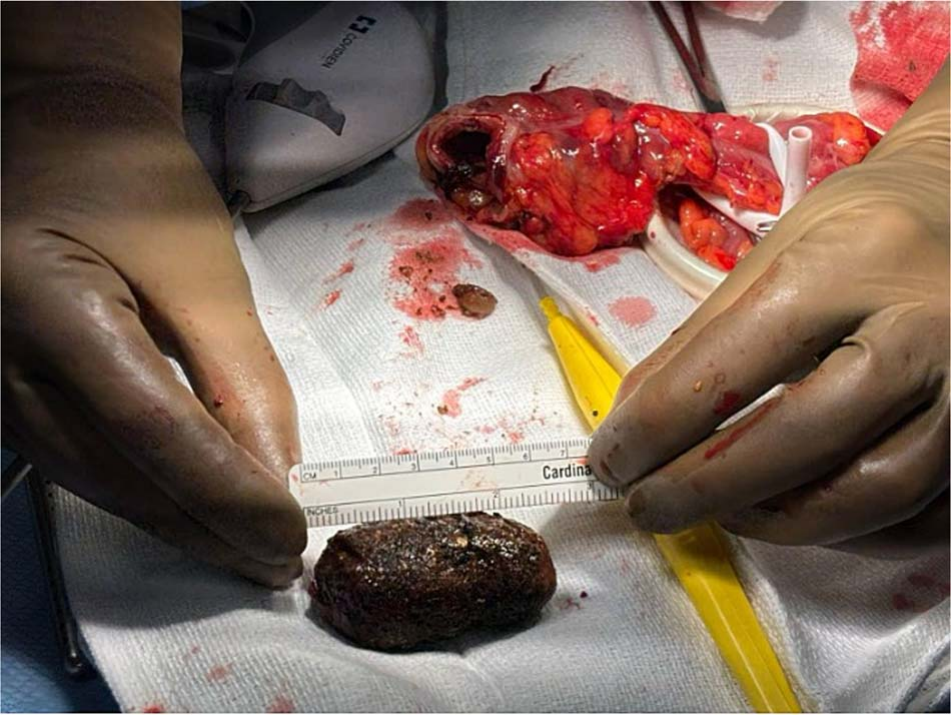
Sigmoid gallstone successfully extracted measuring 6.2 cm.

The patient underwent colonoscopy under general anesthesia for gallstone extraction on the day of admission. After multiple unsuccessful attempts using various endoscopic tools (including a tripod, lasso, and baskets), an exploratory laparoscopy was performed. An intraluminal mass was identified in the sigmoid colon, with visible erosion and ulceration of the external wall. An enterolithotomy was carried out, along with a 10.5 cm segmental resection of the affected sigmoid colon. The gallstone, measuring 6.2 cm, was successfully extracted without complications. The final pathology was consistent with a 6.2 cm gallstone and revealed two adjacent foci of transmural perforation, measuring 0.7 and 0.9 cm in diameter, located in the sigmoid segment. A 4.5 cm mucosal ulceration was also observed, along with evidence of a diverticulitis-related lesion with surrounding inflammatory changes. The patient recovered well and was discharged 4 days postsurgery. At 6-week follow-up, the patient was doing well and reported no biliary symptoms (Figs 1–3).

## Discussion

Gallstone ileus of the colon is a rare cause of large-bowel obstruction, more prevalent among elderly females, caused by migration of a gallstone into the intestinal lumen through a fistula [1, 2]. Sigmoid impaction occurs when the stone lodges in the sigmoid colon, often due to narrowing or pre-existing colonic conditions [2]. Common comorbidities include diverticulosis, cardiovascular disease, and malignancies such as cholangiocarcinoma with liver metastases, endometrial and breast cancer, and esophageal cancer. Clinically, patients typically present with abdominal pain, often accompanied by constipation, vomiting, and abdominal distension [4]. The interval from symptom onset to hospital admission averages ~1 week [2]. Misdiagnosis is frequent, with complications and mortality ranging from 12% to 27% [5]. Radiographic findings of intestinal obstruction and pneumobilia are considered pathognomonic, with plain radiography sensitivity of 40%–70% and CT sensitivity ~93%, making CT the gold standard [5]. CT typically shows small bowel obstruction, an ectopic gallstone, and abnormal gallbladder, while histopathology may reveal ischemic ulceration and perforation [2].

Although nonsurgical management is attempted in many centers, over half of the patients ultimately require surgery [4]. Nonsurgical approaches, including colonoscopic extraction and occasionally adjunctive lithotripsy, are reported mainly for smaller stones [5, 6]. Large stones (>2.5 cm), as can occur after ERCP with sphincterotomy, may still cause obstruction [2, 6].

Definitive management ideally involves enterolithotomy, cholecystectomy, and fistula repair in a single operation to prevent recurrence and fistula-related complications [6]. However, in high-risk patients, enterolithotomy alone is generally safer [6]. In our case, neither cholecystectomy nor fistula repair was performed due to patient factors and surgical risk [3, 5]. Correcting the obstruction alone is considered safe, with minimal complications and possible spontaneous fistula closure [5]. Clinicians should suspect a cholecystocolonic fistula in elderly females with gallbladder lithiasis presenting with colonic obstruction due to its high complication and mortality rates [5]. Enterolithotomy, alone or with segmental resection, is recommended in cases of gastrointestinal erosion or ulceration, though patients remain at risk for recurrent cholecystitis or gallstone ileus. More extensive procedures, such as cholecystectomy with fistula repair, reduce long-term recurrence but carry higher perioperative risk and require intensive postoperative care [3, 7]. The surgical approach should consider patient health, comorbidities, and surgeon experience, with enterolithotomy preferred for high-risk patients and more complex procedures for healthier individuals [7].
